# Decreased inward rectifier potassium current I_K1_ in dystrophin-deficient ventricular cardiomyocytes

**DOI:** 10.1080/19336950.2016.1228498

**Published:** 2016-08-25

**Authors:** Lena Rubi, Xaver Koenig, Helmut Kubista, Hannes Todt, Karlheinz Hilber

**Affiliations:** Department of Neurophysiology and Pharmacology, Center for Physiology and Pharmacology, Medical University of Vienna, Vienna, Austria

**Keywords:** Duchenne muscular dystrophy, dystrophin-associated protein complex, dystrophin-deficient mouse models, I_K1_ inward rectifier potassium current, ventricular cardiomyocytes

## Abstract

Kir2.x channels in ventricular cardiomyocytes (most prominently Kir2.1) account for the inward rectifier potassium current I_K1_, which controls the resting membrane potential and the final phase of action potential repolarization. Recently it was hypothesized that the dystrophin-associated protein complex (DAPC) is important in the regulation of Kir2.x channels. To test this hypothesis, we investigated potential I_K1_ abnormalities in dystrophin-deficient ventricular cardiomyocytes derived from the hearts of Duchenne muscular dystrophy mouse models. We found that I_K1_ was substantially diminished in dystrophin-deficient cardiomyocytes when compared to wild type myocytes. This finding represents the first functional evidence for a significant role of the DAPC in the regulation of Kir2.x channels.

## Introduction

Duchenne muscular dystrophy (DMD), caused by mutations in the gene encoding for the cytoskeletal protein dystrophin, is a severe illness characterized by progressive muscle weakness and degeneration. In affected patients this eventually leads to loss of ambulation, respiratory failure, and premature death. In healthy muscle cells, dystrophin interacts with numerous proteins of the so-called dystrophin-associated protein complex (DAPC),^1,2^ thereby serving as a linker between the cytoskeleton and the extracellular matrix. Disruption of this link in case of dystrophin-deficiency renders muscle tissue vulnerable to mechanical stress.^3,4^

Besides skeletal muscle degeneration, dystrophin-deficiency in DMD also initiates severe cardiac complications. Among those, cardiac arrhythmias and the development of a dilated cardiomyopathy considerably contribute to the morbidity and mortality associated with the disease.^5,6^ Although the precise mechanisms causing these cardiac complications are largely unknown, there is evidence that impaired cardiac ion channel expression and function are involved. For example, dystrophin-deficient ventricular cardiomyocytes derived from mouse models for DMD express less cardiac Na_v_1.5 sodium channel protein than healthy myocytes.^7,8^ Dystrophic cardiomyocytes also show significantly reduced sodium current densities and altered sodium channel gating properties.^7-9^ Reduced sodium currents in murine dystrophic cardiomyocytes match with the impairments in cardiac impulse conduction observed in DMD patients (e.g.^10^). Since ion channels in cardiomyocytes do not function in isolation, but instead in an orchestrated fashion as part of complex protein networks,^11,12^ it is not surprising that dystrophin-deficiency impairs the properties of Na_v_1.5 channels. Na_v_1.5 is considered a member of the DAPC and as such a direct interaction partner of the dystrophin-binding protein syntrophin.^7,13,14^ The lack of dystrophin may therefore disturb regulatory interactions of Na_v_1.5 within the DAPC, which normally are a prerequisite for the proper expression and function of this channel. Accordingly, syntrophin mutations were linked with an abnormal sodium current through Na_v_1.5 channels.^15^

Apart from the well described regulation of Na_v_1.5 sodium channels by the DAPC in cardiomyocytes, evidence has been accumulating that this protein complex also plays a role in regulating Kir2.x inward rectifier potassium channels. Thus, like Na_v_1.5, Kir2.x channels do interact with the DAPC protein syntrophin through their PDZ-binding motifs.^12,16,17^ Moreover, Na_v_1.5 and Kir2.1 colocalize and modulate each other's surface expression in cardiomyocytes,^14,18^ suggesting organization of these 2 channels in a common protein complex. Based on this evidence, the authors of a recently published review article^12^ hypothesized that the DAPC is important in the regulation of Kir2.x channel expression and function. Functional evidence for this hypothesis, however, has been lacking so far. Kir2.x channels in ventricular cardiomyocytes (most prominently Kir2.1^19,20^) account for the inward rectifier potassium current I_K1_, which controls the resting membrane potential, and the final phase of action potential repolarization.^19,21^ Clinically, mutations in the *KCNJ2* gene encoding for Kir2.1 induce diseases associated with severe cardiac arrhythmias and increased risk of sudden cardiac death.^21,22^

In order to test the hypothesis that the DAPC is important in the regulation of Kir2.x channels, here we have studied potential I_K1_ abnormalities in dystrophin-deficient ventricular cardiomyocytes derived from DMD mouse models. Besides the classical dystrophin-deficient mdx mouse,^23^ we also used mice additionally carrying a mutation in the utrophin gene (mdx-utr).^24,25^ The latter DMD mouse model develops a more severe cardiomyopathy with an earlier onset compared to mdx.^25,26^

## Results

**Figure 1.**
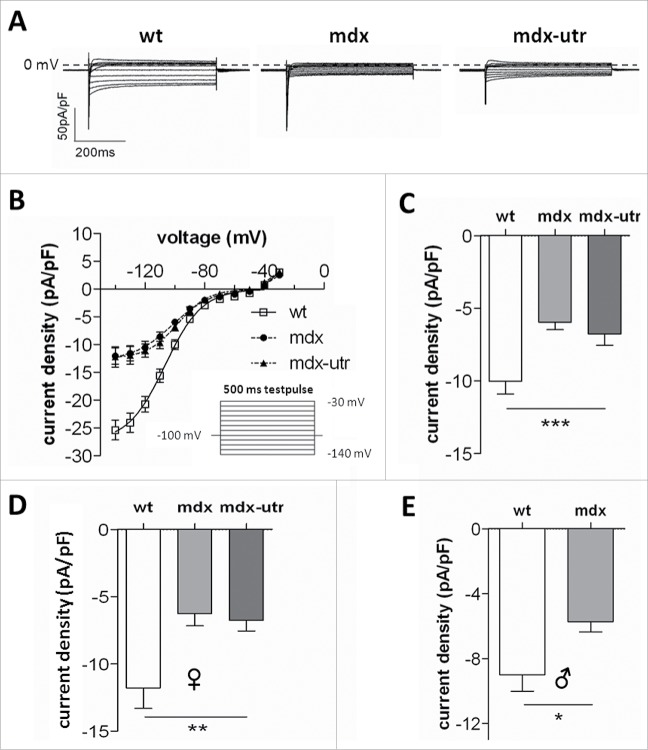
I_K1_ in wt and dystrophic ventricular cardiomyocytes. (A) Typical original potassium current traces of a wt, mdx, and mdx-utr cardiomyocyte elicited from a holding potential of −100 mV by 500-ms steps to various voltages (the pulse protocol is shown in the inset of Fig. 1B). The dashed line indicates the zero current level. (B) Current density-voltage relationships derived from a series of experiments as shown in Fig. 1A (n = 38 for wt, 36 for mdx, and 13 for mdx-utr, respectively). The current levels at the end of the test pulse were evaluated and plotted against the applied voltages. The lines through the data points represent fits with the function: y = Y0/(1 + exp((x-V05)/k)). (C) Current density values at −100 mV (means ± SEM) are compared between wt and dystrophic (mdx and mdx-utr) cardiomyocytes. *** indicates that ANOVA revealed a highly significant difference between the tested groups (p < 0.001). (D) Current density values at −100 mV of wt and dystrophic cardiomyocytes from all experiments on female (♀) mice (n = 14 for wt, 15 for mdx, and 13 for mdx-utr). ** p < 0.01, ANOVA. *E*: Current density values at −100 mV of wt and mdx cardiomyocytes from all experiments on male (♂) mice (n = 24 for wt and 21 for mdx). * p < 0.05 (p = 0.011), Student's t-test.

Figure 1A shows typical original traces of whole cell potassium currents recorded from a wild type (wt) cardiomyocyte, and from dystrophic (mdx and mdx-utr) cardiomyocytes. The currents were elicited by depolarizing and hyperpolarizing voltage steps outgoing from a holding potential of −100 mV (pulse protocol, displayed in the inset of Fig. 1B). A summary of the current density-voltage relationships, derived from a series of such experiments, is presented in Fig. 1B. It can be seen that the current densities of dystrophic cardiomyocytes were substantially decreased when compared with those in wt cells. A comparison of the current densities in wt and dystrophic cardiomyocytes at −100 mV revealed a highly significant difference (Fig. 1C). Figs. 1B and 1C do also show that both types of dystrophic cardiomyocytes displayed similar potassium inward currents. Thus, the current densities of mdx and mdx-utr cardiomyocytes were almost identical over the whole voltage range studied (Fig. 1B). Together these data suggests that dystrophin-deficiency (mdx and mdx-utr) in cardiomyocytes generated the observed decrease in I_K1_ density when compared to the wt, with no additional effect of utrophin-deficiency (only mdx-utr).

In order to check for potential gender differences, the data presented in Fig. 1C are displayed separately with respect to the sex of the animals used for cardiomyocyte isolation (Figs. 1D and 1E). In Fig. 1D the potassium current densities from wt and dystrophic (mdx and mdx-utr) cardiomyocytes derived from female mice only are compared. In Fig. 1E the respective current density data (wt and mdx) from only male mice are presented. Our data imply that dystrophin-deficiency significantly decreased the I_K1_ densities, irrespective of the gender of the animals used for cardiomyocyte isolation.

Taken together, the data presented in Fig. 1 strongly suggest that I_K1_ is substantially diminished in dystrophin-deficient cardiomyocytes.

**Figure 2.**
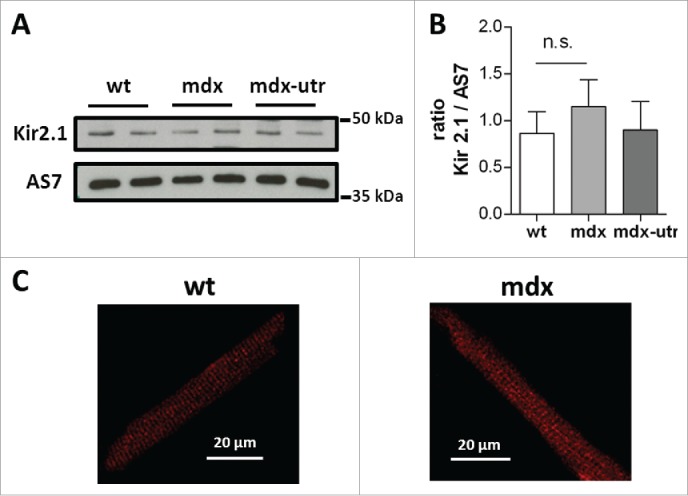
Kir2.1 protein exparession and localization in wt and dystrophic ventricular cardiomyocytes. (A) Representative western blot experiment of membrane lysates from adult wt and dystrophic (mdx and mdx-utr) ventricular tissues stained for Kir2.1 and the β-subunit of a G_s_ protein (AS7). The latter was used as loading control. (B) Relative band intensities of Kir2.1 normalized to the respective band intensities of the loading control plotted as means ± SEM for wt (n = 5) and dystrophic (mdx, n = 6; mdx-utr, n = 2) animals. A Student's t-test did not reveal a significant difference between wt and mdx (p = 0.11; n.s., not significant). (C) Typical examples of Kir2.1 immunostainings of an isolated wt (left) and mdx (right) cardiomyocyte.

Kir2.1 potassium channels are the major determinants of I_K1_ in murine ventricular cardiomyocytes.^19^ In order to test if the decreased I_K1_ in dystrophic cardiomyocytes can be explained by a reduced expression of Kir2.1 channels, we performed western blot experiments with membrane fractions of cardiac ventricular tissues isolated from wt and dystrophic (mdx and mdx-utr) mice. Fig. 2 (A and B) shows that Kir2.1 protein levels in wt and dystrophic ventricles were similar. This suggests that Kir2.1 channel expression is normal in dystrophin-deficient cardiomyocytes. Finally, Fig. 2C compares typical Kir2.1 immunostainings of an isolated wt and a mdx cardiomyocyte. Cross-striations, typical for T-tubular localization of Kir2.1 channels in mouse ventricular cardiomyocytes,^27^ can be observed both in the normal and the dystrophic cell. Similar staining patterns showing cross-striations were obtained in all the studied wt and mdx cardiomyocytes, which were isolated from 3 normal and dystrophic mice, respectively. These immunostaining data implies normal T-tubular Kir2.1 channel localization in dystrophic cardiomyocytes.

## Discussion

Voltage-dependent ion channels in cardiomyocytes are not solely regulated by the transmembrane voltage, but also through interactions with numerous proteins organized in complex networks.^11,12^ Such “ion channel multiprotein assemblies” comprising (but not limited to) anchoring proteins, adaptor proteins, regulatory proteins, and enzymes regulate the trafficking, expression, localization, and function of an associated channel.

A recently published review article^12^ deals with protein assemblies of cardiac Na_v_1.5 sodium and inward rectifier Kir2.1 potassium channels, that control cardiac excitability and, in case of dysregulation, introduce arrhythmogenesis. Interestingly, these 2 channels not only share a number of common protein interaction partners (e.g., caveolin-3, synapse-associated protein-97, and syntrophin) (^12^ and references therein), but also modulate each other's cell-surface expression.^14,18^ These findings suggest organization of Na_v_1.5 and Kir2.1 in one or several common protein complexes. Here the DAPC stands out as a likely candidate. Thus, at the cardiomyocyte membrane, both channels interact through the PDZ domain-scaffolding protein syntrophin (see Fig. 2A in^12^), which is a direct binding partner of dystrophin within the DAPC.

The well-known fact that disruption of the DAPC (due to dystrophin-deficiency) in cardiomyocytes significantly impairs Na_v_1.5 channel expression^7,8^ and function^7-9^ suggests that an intact DAPC is required to maintain normal Na_v_1.5 channel properties. In contrast, the notion that the DAPC is also important in the regulation of Kir2.x channel expression and function^12^ has remained purely speculative till this day. In the present study, we provide the first functional evidence that the DAPC indeed also impacts Kir2.x channels by showing that I_K1_ in dystrophin-deficient cardiomyocytes is substantially diminished. This suggests that, like Na_v_1.5 channels, Kir2.x channels require interaction with the DAPC for their proper functioning. In both cases, a disruption of dystrophin leads to a marked reduction in the respective current (I_Na_ and I_K1_, respectively). A reduced I_K1_ due to dystrophin-deficiency may provide an explanation for the low resting potentials measured in dystrophic cardiac^8,28^ and skeletal^29-31^ myocytes when compared to their healthy counterparts. It also represents a so far unknown potential mechanism to cause cardiac arrhythmias in DMD patients.

An obvious difference between the regulatory action of the DAPC on Na_v_1.5 and Kir2.x channels in cardiomyocytes, which has emerged from our findings and previous studies, concerns the effect of the protein complex on channel expression. Thus, dystrophin-deficiency induces a substantial reduction in Na_v_1.5 channel expression,^7,8^ but has no (the present study) or only minor^7^ effect on Kir2.1 protein levels. This suggests that the decreased I_K1_ in dystrophin-deficient cardiomyocytes is not due to reduced Kir2.1 channel expression, but is caused by another mechanism. Because Kir2.1 channel localization in dystrophic cardiomyocytes may also be normal (Fig. 2C), channel inhibition by cytoplasmic regulatory factors is likely. Follow-up studies are needed to identify the responsible regulator(s). A better insight in how the DAPC regulates Kir2.x channels to control cardiac excitability, and how mutations in genes encoding for the involved proteins impair this regulation should increase our understanding of diseases associated with arrhythmias such as DMD, and may lead to improvements in therapy. Finally, DAPC regulation of inward rectifier potassium channels may also be relevant in other organs, e.g. the brain. Thus, in brains from mdx mice, dystrophin-deficiency was associated with a significant reduction in Kir4.1 mRNA expression and protein content.^32^

## Materials and methods

The study conforms to the guiding principles of the Declaration of Helsinki and coincides with the rules of the University Animal Welfare Committee.

### Mouse models

Wild type (wt) C57BL/6 mice, dystrophin-deficient mdx,^23^ and dystrophin/utrophin-deficient double mutant mdx-utr^24,25^ mice, backcrossed on the C57BL/6 background for more than 12 generations, were used for the study. Details about the mutations are described in our earlier work.^33^ Through the text, the dystrophin−/− status (utrophin+/+) is named “mdx,“ and double mutant mice (dystrophin−/− and utrophin−/−) are termed “mdx-utr.” The mice were genotyped using standard PCR-assays. For comparisons between mdx and mdx-utr, littermates were used.

### Isolation of ventricular cardiomyocytes

15–25 week-old female mice (2 wt, 2 mdx, and 2 mdx-utr animals) and male mice (3 wt and 3 mdx animals) were killed by cervical dislocation, and cardiomyocytes were isolated from the ventricles of their hearts by using a Langendorff setup as described in our previous work.^9^

### I_K1_ measurements

Applying the whole cell patch clamp technique and established pulse protocols, I_K1_ was recorded at room temperature (22 ± 1.5°C) from ventricular cardiomyocytes up to 8 hours after preparation using an Axoclamp 200B patch clamp amplifier (Axon Instruments, Union City, CA). Pipettes were formed from aluminosilicate glass (AF150-100-10; Science Products, Hofheim, Germany) with a P-97 horizontal puller (Sutter Instruments, Novato, CA). Their resistances were between 0.8 and 2 MΩ when filled with pipette solution (see below). Data acquisition was performed with pClamp 6.0 software (Axon Instruments) through a 12-bit A-D/D-A interface (Digidata 1200; Axon Instruments). Data were low-pass filtered with 1–10 kHz (−3 dB) and digitized at 10–100 kHz. Data analysis was performed using Clampfit 10.2 (Axon Instruments) and GraphPad Prism 5.01 (San Diego, USA) software. The cells were bathed in 140 mM NaCl, 4 mM KCl, 2 mM CaCl_2_, 2 mM MgCl_2_, 5 mM HEPES, 5 mM Glucose, pH = 7.4 (adjusted with NaOH). The pipette solution contained 10 mM NaCl, 140 mM KCl, 2 mM EGTA, 1 mM MgCl_2_, 0.1 mM Na-GTP, 5 mM Mg-ATP, 10 mM HEPES, pH = 7.2 (adjusted with KOH). Potassium currents were elicited by 500-ms hyper- and depolarizing voltage steps between −140 and −30 mV from a holding potential of −100 mV (see inset in Fig. 1B). Around the resting membrane potential and at more hyperpolarized voltages, the ventricular I_K1_ conductance is much larger than that of any other potassium current.^21^ In murine ventricular cardiomyocytes, currents through Kir2.1 channels represent the major component of I_K1_ consistent with the finding that myocytes isolated from Kir2.1−/− mice completely lacked detectable whole cell I_K1_ in 4 mM external potassium.^19^ A potential “contamination” of I_K1_ by other inward rectifying potassium currents (I_K,ATP_,^34^ I_K,ACh_,^35^ and I_K,Ca_^35^) in our experiments was excluded by the composition of our experimental solutions. Thus, I_K,ATP_ was inhibited by the presence of mM concentrations of ATP in the pipette solution,^36,37^ and activation of I_K,ACh_ was prevented by the lack of acetylcholine. I_K,Ca_ activation could be excluded because calcium channels do not activate at potentials more negative than −60 mV at which significant I_K1_ was detectable. Further, calcium was absent from the pipette solution which additionally contained 2 mM of the calcium buffer EGTA (see above). For the determination of I_K1_ density-voltage relations, the current amplitudes at the end of the test pulses to various potentials were measured. These were then divided by the cell capacitance to obtain current densities. I_K1_ recordings from both wt and dystrophic cardiomyocytes were always started 5 min after whole cell access was attained to avoid potential artifacts due to time-dependent shifts in current properties.

### Western blotting

The ventricles from 5 wt (3 female, 2 male), 6 mdx (3 female, 3 male), and 2 mdx-utr (both female) mouse hearts were used for the western blot experiments. Membrane proteins were isolated using the following protein isolation protocol carried out at 4°C. Frozen cardiac ventricles were cut into 15 μm slices at −20°C using a cryoslicer. Samples were weighed, blood was removed by washing the samples with ice cold PBS, and solution A (10 mM HEPES, pH = 7.4, 10% sucrose, 5 mM EDTA, 1 mM Dithiothreitol (DTT), 2 μg/ml aprotinin, 10 μg/μl leupeptin, 1 mM pefablock) was added (4 μl of solution A / mg of tissue). The tissues were homogenized on ice. After gentle centrifugation to remove bigger tissue pieces, samples were centrifuged at 1000 x g to separate the nuclei from the cytosol and the membrane protein fraction. In the next step the supernatant was centrifuged for 45 min at 100.000 x g to separate the cytosolic protein fraction from the membrane protein fraction. Membrane proteins were re-suspended in 200 μl of solution B (10 mM HEPES, pH = 7.4, 10 % sucrose, 2 mM EDTA, 1 mM DTT, 2 μg/ml aprotinin, 10 μg/μl leupeptin, 1 mM pefablock), and protein concentrations of the lysates were determined by spectrophotometry. The membrane protein lysates of equal protein concentrations were blotted on a 9% sodium dodecyl sulfate polyacrylamide gel electrophoresis (SDS-PAGE) for 80 to 90 min at a voltage of 150 V. Separated proteins were transferred to a nitrocellulose membrane (GE Healthcare, United Kingdom) using the wet transfer method for 50 min on ice. The membrane was blocked for 2 hours at room temperature using 5% bovine serum albumin (BSA) and incubated over night with the primary antibody (anti-Kir2.1 polyclonal antibody produced in rabbit; APC-026, Alomone Labs, 1:1000 in 2% BSA) at 4°C. On the next day, after 3 PBS washing steps, the membrane was incubated with the secondary antibody (anti-rabbit IgG, horse radish peroxidase-linked antibody; 7074, Cell Signaling Technology, 1:10000) at room temperature for 60 min. The protein bands were visualized using SuperSignal Western Pico Chemiluminescent Substrate (Thermo Scientific, USA) and ECL hyperfilms (GE Healthcare, United Kingdom). The protein band intensities were measured using ImageJ software (http://rsbweb.nih.gov/ij) for western blot analysis. To subtract background signals, the specific intensity peaks were defined, and the area under the curve was normalized to the area under the curve of the loading control protein intensities. An antibody (AS7) recognizing the β1/β2 subunit of membrane bound G_S_ proteins was used as loading control (AS7 was generated by M. Hohenegger;^38^ AS7 complies with the original non-specific antiserum K521, and was raised against a peptide enclosing residues 8–23 in the sequence of the beta1/beta2 subunit). Western blot experiments were performed several times per heart lysate. The obtained values for every lysate were averaged. These averaged values (resulting in n = 5 for wt, n = 6 for mdx, and n = 2 for mdx-utr) were used for the statistical analyses, whereby only wt and mdx were compared.

### Immunostaining

After the Langendorff cell isolation procedure the ventricular cardiomyocytes were plated on cover slips, and, 90 min later, fixed in 3.5% paraformaldehyde for 10 min. Cell culture medium was removed, the cells were washed 3 times with PBS, permeabilized in 0.1% Triton X-100 for 5 min at room temperature, and washed again 3 times with PBS. This was followed by a 2 hour block with 10% horse serum and 0.01% azide in PBS. Thereafter, the cells were incubated with Anti-Kir2.1 antibody (APC-026, Alomone Labs; 1:500 in PBS) at 4°C overnight. On the next day, the cells were washed 3 times with PBS, and incubated for 60 min with the corresponding secondary antibody (Alexa Fluor 594, #A21207, Invitrogen; 1:500 in PBS) at room temperature. Subsequently, after 3 more PBS washing steps, the cells were mounted, dried and stored at 4°C. The slides were finally analyzed using a LSM 510 confocal microscope (Zeiss, Jena, Germany). For the immunostaining experiments 3 wt and 3 mdx mice (15–25 week-old) were used for cell isolation.

### Statistical analyses

Data represent means ± SEM. Statistical comparisons between wt, mdx, and mdx-utr were made using oneway ANOVA (for independent samples, GraphPad Prism Software, La Jolla, USA). In case only 2 groups had to be compared, an unpaired 2-tailed Student's t-test was performed. A p value < 0.05 was considered significant.

## Abbreviations

DAPC: dystrophin-associated protein complex
DMD: Duchenne muscular dystrophy.
